# Influence of Sintering Process Conditions on Microstructural and Mechanical Properties of Boron Carbide Ceramics Synthesized by Spark Plasma Sintering

**DOI:** 10.3390/ma14051100

**Published:** 2021-02-26

**Authors:** Yingying Liu, Sheng Ge, Yihua Huang, Zhengren Huang, Deku Zhang

**Affiliations:** 1 State Key Laboratory of High Performance Ceramics and Superfine Microstructure, Shanghai Institute of Ceramics, Chinese Academy of Sciences, Shanghai 201800, China; liuyingying@student.sic.ac.cn (Y.L.); gesheng@student.sic.ac.cn (S.G.); 2 College of Materials Science and Opto-Electronic Technology, University of Chinese Academy of Sciences, Beijing 100049, China; 3 School of Materials Science and Engineering, Nanjing University of Science and Technology, Nanjing 210094, China

**Keywords:** spark plasma sintering, B_4_C ceramics, sintering conditions, microstructure, mechanical properties

## Abstract

Boron carbide (B_4_C) ceramics were synthesized by spark plasma sintering at a temperature between 1600 and 2050 °C without employing any sintering additives. The effect of sintering process parameters, such as temperature, holding time, pressure, hearting rate, and pulsed electric current, and the particle size of the raw powder on the densification behavior and mechanical properties of B_4_C ceramics, were comprehensively and systematically investigated. Hardness and fracture toughness of B_4_C that has a density close to the theoretical value were found to be 33.5 ± 0.2 GPa and 3.21 ± 0.13 MPa·m^1/2^, respectively. Electron backscatter diffraction (EBSD) analysis revealed no abnormal growth of grains due to an increase in holding time and pressure. Twin structures present in ceramics are beneficial for their mechanical performance.

## 1. Introduction

Boron carbide (B_4_C) is an excellent candidate material in many high-performance fields due to its fantastic properties such as low density (2.52 g/cm^3^), high melting point, high level of hardness (after diamond and cubic boron nitride), fine chemical stability, good wear resistance, and wonderful neutron absorption capacity [1,2,3]. Due to these excellent properties, B_4_C ceramics have been used in many different fields including cutting tools, grinding wheels, blasting nozzles, lightweight armors, neutron shielding materials, high-temperature-resistance wear parts, etc [4,5,6]. However, the full theoretical density of B_4_C ceramics is difficult to achieve because of the low diffusion coefficient [4,7] caused by a strong covalent bond between B and C, and due to retardation of the densification process by B_2_O_3_ present on the surface of B_4_C [8,9].

Numerous attempts have been made to improve the sintered density of B_4_C ceramics [7,8,9,10]. Temperature near the melting point is necessary for the pressureless sintering of the B_4_C ceramics. Such a high temperature can lead to grain coarsening, thereby reducing the performance of B_4_C ceramics. At the same time, pressureless sintering can produce B_4_C ceramics with a relative density of 90% along with pores that can further reduce the performance of B_4_C ceramics [11,12]. To reduce the sintering temperature of B_4_C ceramics, many sintering additives (C, Ti, Cr, Ni, Cu, TiB_2_, TiC, Al_2_O_3_, etc.) have been added to accelerate the densification process [13,14,15,16,17]. The addition of such sintering additives can cause other impurity phases in the final product. The high purity of B_4_C ceramics is essential in many different applications, particularly in the nuclear field [1,2].

Hot pressing sintering (HPS) and spark plasma sintering (SPS) are two effective methods for structural ceramic materials. Compared to HPS, SPS can produce materials with uniformly refined particles and has excellent performance under fast heating speed, low sintering temperature, and short holding time, which makes it very popular amongst researchers [18]. SPS has been widely used to sinter B_4_C ceramics, however, the majority of reports have limited the investigation to understand the effect of a few parameters on ceramic properties. Li et al. [7] mainly explored the influence of different sintering temperatures on the relative density and microstructure of B_4_C ceramics. Finally, at 1600 °C and a heating rate of 100 °C/min, a B_4_C ceramic with a density of 98.33%, a hardness of 31 GPa, and a toughness of 2.66 ± 0.29 MPa·m^1/2^ was obtained. Therefore, systematically exploring the influence of SPS parameters on the densification and performance of B_4_C ceramics is of great significance, and it can also guide the sintering of B_4_C-based composite ceramics.

In this work, the influence of SPS parameters (sintering temperature, holding time, sintering pressure, heating rate, pulse rate) and particle size of the starting material on the performance of B_4_C ceramics was systematically investigated. The grain size distribution and orientation distribution of the samples were characterized by EBSD. The influence of the microstructure on mechanical properties is also discussed.

## 2. Experiment

### 2.1. Characterization of Raw Powders

**Figure 1:**
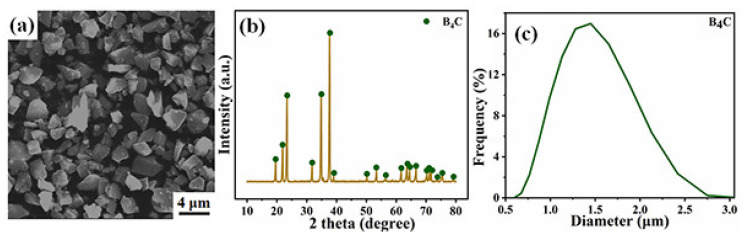
(**a**) Scanning electron microscopy (SEM) image, (**b**) X-ray diffraction (XRD) spectrum, and (**c**) particle size distributions of B_4_C powders. Adapted with permission from ref. [19]. 2020 Yingying Liu.

Commercially available micron-sized powders of B_4_C (d_50_ = 1.47 μm, Aladdin, Shanghai, China) were used as the raw material. The scanning electron microscopy (SEM, Magellan 400, FEI, Hillsboro, OR, USA) picture, X-ray diffraction (XRD, D/Max-2250V, Rigaku, Tokyo, Japan) spectra, and particle size distribution are shown in Figure 1.

### 2.2. Sintering

B_4_C powders were put in a graphite die with a diameter of 20 mm, where a graphite foil separated the powders and the die in order to facilitate the demolding after sintering. Subsequently, the graphite mold filled with raw powder was put in the SPS chamber and sintered in accordance with the set procedure. The densification process of B_4_C ceramics took place between 1800 and 2050 °C. The effect of holding time, pressure, and different pulsed electric currents on the densification, microstructural, and mechanical performance of B_4_C ceramics was explored.

### 2.3. Characterization

In order to remove the graphite foil from the surface, the B_4_C samples were simply ground on a grinding machine. The densities of the ceramics were calculated by the Archimedes principle. The Vickers hardness and the fracture toughness of the sintered B_4_C ceramics were determined by the indentation method by applying 9.8 N for 10 s. The toughness was measured by following the approach of Anstis et al. [20,21] and the equation is given below:

(1) $$K_{IC}=0.016{\left(E/H\right)}^{1/2}\times P/c^{3/2}$$

where *E* is the Young’s modulus, *H* is the Vickers hardness, *P* is the applied load and *c* is the half-length of the indentation crack. The indentation tests were performed on the surface polished to 0.5 μm with diamond paste. The average of the results of the six tests was used as the final hardness and toughness result. The characteristics of the grain boundaries were analyzed using transmission electron microscopy (TEM, JEM-2100F, JEOL, Tokyo, Japan). EBSD (Magellan 400, FEI, Hillsboro, OR, USA) was used to obtain the grain size distribution and orientation of B_4_C ceramics.

## 3. Results and Discussion

### 3.1. Influence of Sintering Parameters on the Microstructural and Mechanical Properties of B_4_C Ceramics

The microstructural and mechanical properties of the synthesized B_4_C ceramics were studied at different sintering process conditions to understand the influence of sintering temperature, soaking time, applied pressure, heating rate, pulsed electric current, and particle size of the raw powders.

#### 3.1.1. Influence of Sintering Temperature

**Figure 2:**
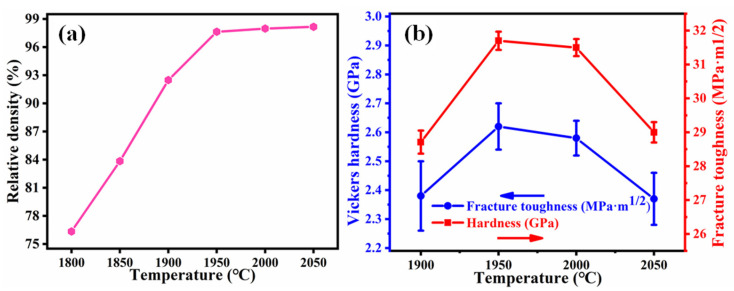
(**a**) Relative density of B_4_C ceramic as a function of temperature; (**b**) Hardness and fracture toughness of B_4_C ceramic as a function of temperature.

**Figure 3:**
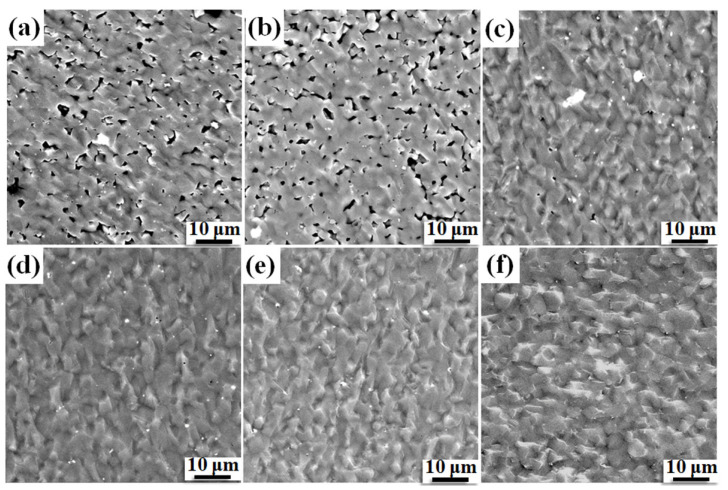
SEM morphologies of fractured surfaces sintered at different temperatures: (**a**) 1800 °C, (**b**) 1850 °C, (**c**) 1900 °C, (**d**) 1950 °C, (**e**) 2000 °C, (**f**) 2050 °C.

Figure 2a shows the bulk densities of B_4_C ceramics sintered at different temperatures. The relative density increases linearly from 76.3% to 98.1% with an increase in temperature from 1800 to 1950 °C and shows saturation behavior upon further increase in temperature above 1950 °C. This can further be confirmed from the SEM images of the fractured surfaces (Figure 3). Many holes can be observed in Figure 3a,b, which indicates that the relative density is low. The number of holes is reduced significantly, as shown in Figure 3c, which is an indicator of increasing relative density. Holes are not evident in Figure 3d–f, which demonstrates that the samples have a high relative density. The fracture surfaces of B_4_C ceramics are smooth and flat, exhibiting the characteristics of transgranular fracture. Figure 2b shows the hardness and toughness of B_4_C ceramics measured at different temperatures. The hardness and fracture toughness increase initially and then decrease with increasing temperature. The hardness and toughness reach their maximum values at 1950 °C, that is, 31.70 ± 0.27 GPa and 2.62 ± 0.05 MPa·m^1/2^, respectively. Zhang et al. [18] produced B_4_C ceramics with a relative density of over 94% and a Vickers hardness of 26.4 GPa by SPS at 1900 °C. The hardness and toughness increase with an increase in the relative density at temperatures between 1900 and 1950 °C, which demonstrates that the relative density plays a leading role in this temperature range. On further increase in temperature to 2050 °C, the density and mechanical performance of the sample decline, which may be due to the growth of part of the grains, or the change of the grain shape (from equiaxed crystals to long, rod-shaped crystals). The specific explanation needs to be further analyzed in conjunction with the microstructure. The sintering temperature further affects the mechanical properties of the sample by affecting its relative density and microstructure. When the sintering temperature is changed from low to high temperature, the sample is compacted quickly to achieve better performance. If the temperature is increased, the grain size may increase slightly, or the grain shape may change or side reactions may occur, which is not conducive to further performance optimization; at the same time, too high of a sintering temperature places higher requirements on the equipment and increases production costs. Therefore, the optimal sintering temperature is 1950 °C.

#### 3.1.2. Effect of Holding Time

**Figure 4:**
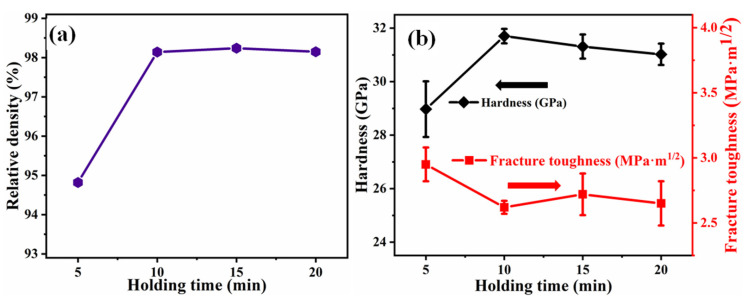
(**a**) Relative density of B_4_C ceramics (sintered at 1950 °C) as a function of holding time; (**b**) Hardness and fracture toughness of B_4_C ceramics as a function of holding time.

Figure 4a displays the variation of the relative density of B_4_C ceramics sintered at 1950 °C with respect to the holding time. The relative density is found to be increased from 94.8% to 98.1% with an increase in the soaking time from 5 to 10 min. Above 10 min, the relative density shows saturating behavior and becomes constant at about 98%. The hardness and toughness of B_4_C samples obtained at 1950 °C are depicted in Figure 4b. Hardness increases as the holding time increases from 5 to 10 min due to the increase in density. However, the fracture toughness exhibits a decreasing trend as the holding time increases. In some cases, the existence of holes can cause a false increase in toughness. The hardness and toughness do not change and show saturation behavior around the highest value as the holding time increases further from 10 to 20 min. Theoretically, the density of the sample increases with the extension of the holding time, but when the holding time is too long, on the one hand, part of the crystal grains may be coarsened; on the other hand, the shape of some crystal grains may change, which may lead to the density of the sample and performance being slightly reduced. The behavior of the density, hardness, and toughness with respect to the holding time suggests that the optimized holding time is 10 min.

**Figure 5:**
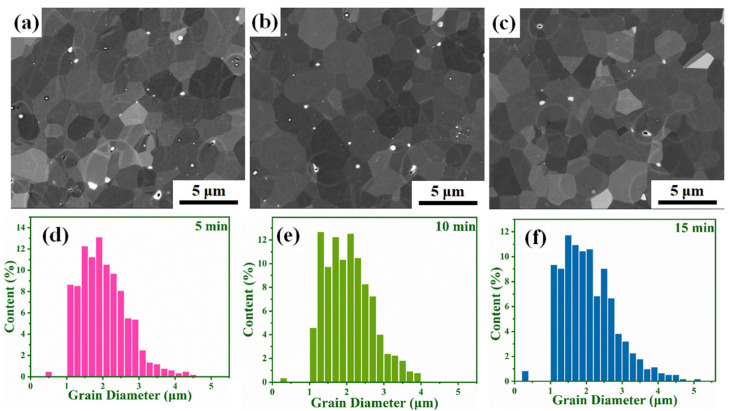
SEM images and Grain size frequency of samples sintered at 1950 °C with different holding times: (**a**,**d**) 5 min, (**b**,**e**) 10 min, and (**c**,**f**) 15 min.

**Figure 6:**
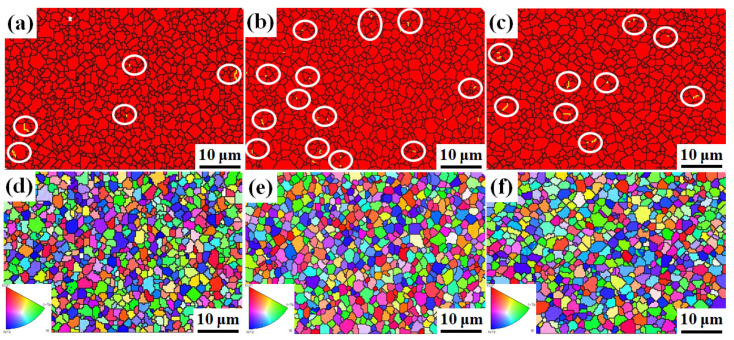
Grain orientations of B_4_C ceramics sintered at 1950 °C with different holding times: (**a**,**d**) 5 min, (**b**,**e**) 10 min, and (**c**,**f**) 15 min.

The B_4_C ceramics have equiaxed grains with clear grain boundaries, which can be observed in the SEM pictures shown in Figure 5a–c. The sample shown in Figure 5a contains more pores than Figure 5b,c, which is similar to the calculated value of the relative density. No obvious grain size coarsening is observed. In Figure 5c, rod-shaped crystals can be observed, indicating that the shape of the crystal grains changes from equiaxed crystals to rod-shaped crystals over time. The variation of grain size of B_4_C ceramics fabricated at 1950 °C with different holding times is displayed in Figure 5d–f. The size of the grains is mostly between 1 and 4 μm. The average grain sizes of the B_4_C ceramics for holding times of 5, 10, and 15 min are 2.02, 2.06, and 2.07 μm, respectively. The increasing trend of the average grain size with respect to the holding time confirms that B_4_C ceramics produced by SPS have no abnormal grain growth. At the same time, it can be seen in the grain size distribution histogram that the number of grain sizes between 4 and 5 μm in Figure 5f is significantly more than that in Figure 5d,e. This indicates that as the holding time increases, some of the grains in the sample do grow in size. Therefore, as the holding time continues to extend, the density and performance of the sample decrease slightly. Figure 6a–c shows the distribution of the B_4_C phase in samples produced at different holding times. The red areas represent B_4_C grains and the yellow lines indicate twin boundaries. B_4_C has smaller grains. Many twin boundaries are observed in Figure 6b,c. The twin boundaries of SPS-produced B_4_C ceramics have also been reported by others [22,23,24]. The discussion on the twin boundaries will be carried out in the next part. Figure 6d–f shows the grain orientation of the samples at different holding times. Different colors correspond to different grain orientations. It can be seen that the B_4_C ceramics produced by SPS at different holding times have no obvious grain orientations.

#### 3.1.3. Effect of Sintering Pressure

**Figure 7:**
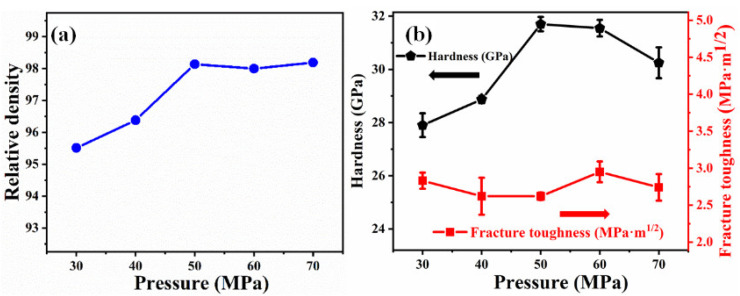
(**a**) Relative density of B_4_C ceramics (sintered at 1950 °C for 10 min) as a function of pressure; (**b**) Hardness and fracture toughness of B_4_C ceramics as a function of pressure.

Figure 7a illustrates the effect of sintering pressure on the relative density of B_4_C ceramics. The relative density of the sample improves from 95.5% to 98.1% as the applied pressure increases from 30 to 50 MPa. Upon further increase in the pressure above 50 MPa, the relative density shows saturating behavior. Figure 7b shows the variation of Vickers hardness and toughness of the samples obtained at 1950 °C with pressure. As the applied pressure increases, the hardness increases up to 50 MPa, and then decreases upon a further increase in the applied pressure. The increase in hardness is mainly because of the improvement of the density of ceramics up to 50 MPa. The decreasing behavior of the hardness above 50 MPa could be attributed to the coarsening of some grains caused by increasing pressure. At a pressure of 30 MPa, the samples show high toughness due to the low density (only 95.5%) caused by the presence of many pores. When the density of the samples is stable at about 98%, the fracture toughness becomes stable. The effect of applied pressure is similar to that of sintering temperature and holding time. When the sintering pressure is increased from a small amount, the density and performance of the sample are improved. When the applied pressure continues to increase, it may cause the sample to become over-sintered, which will change the shape of some grains and affect its performance.

**Figure 8:**
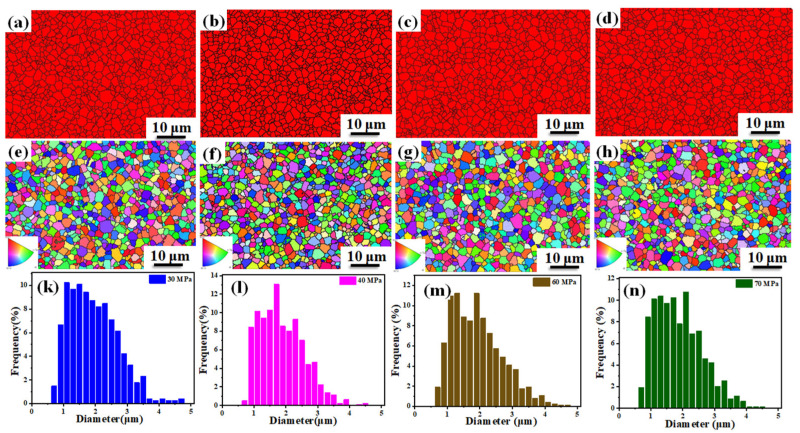
Electron backscatter diffraction (EBSD) of B_4_C ceramics sintered at 1950 °C for 10 min with different pressures. 30 MPa (**a**,**e**,**k**), 40 MPa (**b**,**f**,**l**).60 MPa (**c**,**g**,**m**), 70 MPa (**d**,**h**,**n**).

The grain distribution of the B_4_C phase is illustrated in Figure 8a–d. The size of grains corresponding to the B_4_C phase is small and no abnormal growth is observed. The orientation of B_4_C grains is shown in Figure 8e–h in which different colors represent different orientations. The grain growth of B_4_C ceramics sintered under different pressures has no preferred orientation. The grain size distributions of B_4_C ceramics are shown in Figure 8k–n, which are mostly between 1 and 4 μm. With the increase in pressure, the number of grains that have a size between 3 and 4 μm increases slightly. This shows that when the applied pressure continues to increase, the size of some grains does increase, which makes the performance of the sample slightly lower.

#### 3.1.4. Influence of Heating Rate

The relative density of B_4_C ceramics obtained at different heating rates is illustrated in Figure 7a. To observe the influence of the heating rate on the relative density effectively, the temperature of 1850 °C was selected (1850 °C was also selected to research the influence of the pulse rate). As the heating rate increases, the density of the B_4_C samples increases to 100 °C/min and then decreases above 100 °C/min. The highest relative density is obtained at the heating rate of 100 °C/min. At slower heating rates of 50 and 75 °C/min, there could be unwanted reactions that could affect the diffusion of the substance and ultimately lead to a comparatively low relative density. As the heating rate increases beyond 100 °C/min up to 250 °C/min, the density of the B_4_C ceramics decreases. At higher heating rates, the time is not sufficient for mass transport. The released gas does not have enough time to leave the system. Residual gas could cause closed pores in the sintered sample, which would prevent improvement in the relative density and mechanical performances. Although a slow heating rate is conducive to the diffusion and transportation of substances, it will also cause some undesirable reactions to occur. If the heating rate is too fast, the substance transportation will be too late to proceed, and it will not be conducive to the increase in density. Based on experimental results, the best heating rate is 100 °C/min.

#### 3.1.5. Effect of Pulse Rate

**Figure 9:**
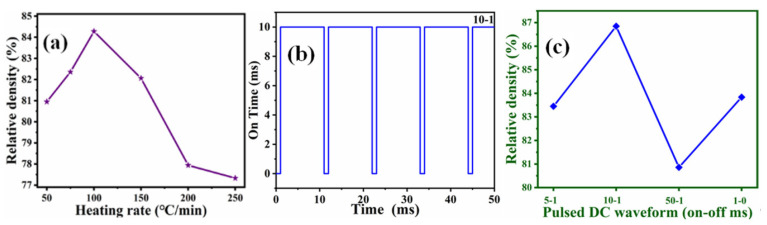
(**a**) Relative density of samples as a function of heating rate; (**b**) Schematic of pulsed DC waveform showing 10–1 (ms–ms) pulse sequence; (**c**) Relative density of samples as a function of pulsed DC waveform.

DC pulse voltage provides power for material transport [25,26,27,28,29,30]. It is significant to explore the influence of different pulse rates on the relative density of samples. Figure 9b represents a schematic of the pulsed DC waveform showing a 10–1 (ms–ms) pulse sequence. Figure 9c illustrates the influence of pulse rate on the relative density of B_4_C ceramics. The density reaches the highest value in the 10–1 (ms–ms) pulse sequence. It is preliminarily concluded that the effect of the pulse rate on the densification of the ceramic is manifested in the acceleration of the material diffusion in the sample when the pulse is turned on; in the rapid increase of the densification when the pulse is turned off, the rate of the material diffusion process is reduced, which facilitates the elimination of the generated gas and the escape of bubbles in the sample. Such a suitable pulse rate can ensure a higher level of gas removal and densification process, which ultimately manifests as a higher density of the sample.

#### 3.1.6. Effect of the Particle Size of the Raw Material

**Figure 10:**
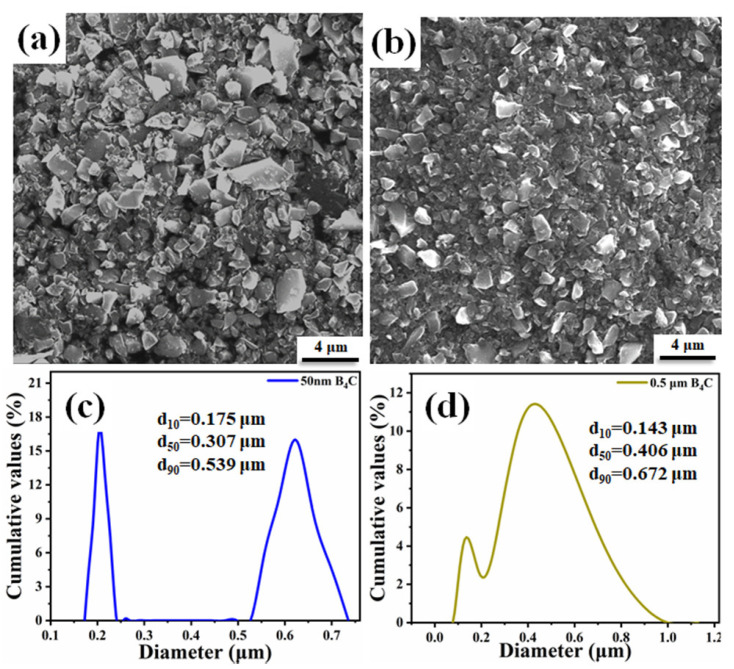
Characteristics of B_4_C powders: (**a**,**c**) A-B_4_C; (**b**,**d**) B-B_4_C.

The raw material powder used in the above experiment is B_4_C powder with d_50_ = 1.47 μm (named as B_4_C). In this part of the work, commercially available 50 nm (named as A-B_4_C) and d_50_ = 0.406 μm (named as B-B_4_C) B_4_C powder was selected for sintering to explore the effect of the particle size of the raw material on the density and mechanical performance of the B_4_C ceramics. Figure 10 shows the SEM morphologies and particle size distributions of starting powders. As indicated in Figure 10c, the A-B_4_C powder presents an obvious bimodal distribution; some of the particles are very small, with a size of about 0.2 μm, and some of the particles are larger, mainly distributed at about 0.6 μm, indicating that some particles are agglomerated.

**Figure 11:**
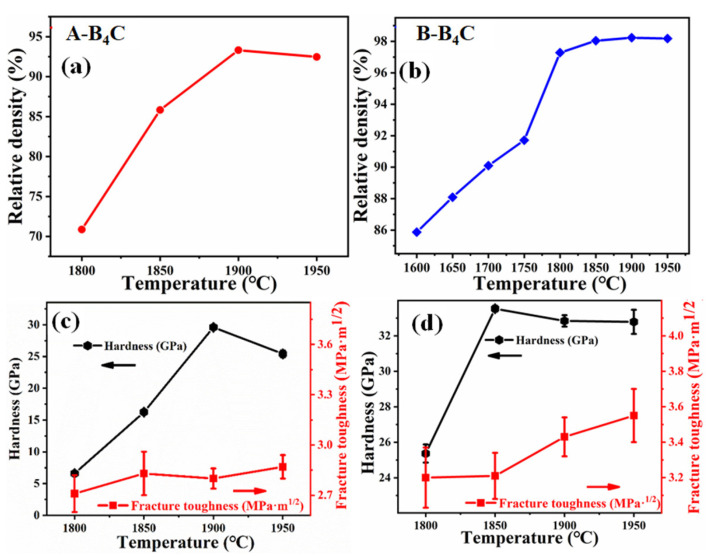
Relative density (**a**,**b**) and mechanical properties(**c**,**d**) of B_4_C ceramics sintered at different temperatures.

The relative density of A-B_4_C powder sintered at different temperatures is depicted in Figure 11a. The relative density enhances as the temperature increases and attains a maximum of 93.2% at 1900 °C and then decreases slightly upon a further increase in temperature. The variation of the hardness and toughness of the sample with temperature is illustrated in Figure 11c. The hardness improves with an increase in temperature up to 1900 °C and then decreases with a further increase in the temperature. The relative density of the B_4_C ceramics mainly affects such behavior. The fracture toughness value of the sample is between 2.7 and 2.9 MPa·m^1/2^. The variation is not significant, which could be due to the effect of the holes presented in the B_4_C ceramics.

**Figure 12:**
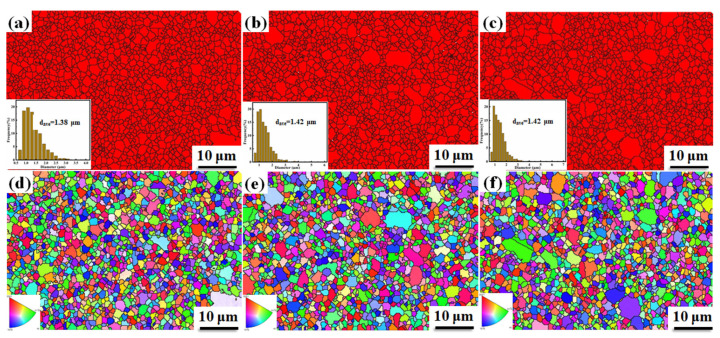
The grain size and orientation of B-B_4_C sintered at different temperatures: (**a**,**d**) 1850 °C, (**b**,**e**) 1900 °C, and (**c**,**f**) 1950 °C.

Figure 11b shows the density of B-B_4_C powder fabricated at various temperatures. The density improves as the temperature increases. The density of the B-B_4_C ceramics sintered at 1850 °C is close to 98%. Above 1850 °C, the relative density increases slightly. Thus, 1850 °C is a suitable sintering temperature. The hardness and toughness of the samples are illustrated in Figure 11d. The hardness increases first and then stabilizes as the temperature increases from 1800 to 1950 °C. The B_4_C ceramic with the highest hardness of 33.5 ± 0.2 GPa was obtained at 1850 °C and the toughness was 3.21 ± 0.13 MPa·m^1/2^. The comprehensive mechanical performances of the B_4_C ceramics are better than those in previous reports [18,22,24,31]. The relevant results are shown in Table 1. The grain size distribution and grain orientation of B-B_4_C ceramics sintered at various temperatures are illustrated in Figure 12. The average grain sizes of B_4_C ceramics sintered at different sintering temperatures (1850, 1900, and 1950 °C) are 1.38, 1.42, and 1.42 μm, respectively. The mechanical properties of the samples are affected not only by the relative density but also by the grain size. The refinement of grain size can optimize the properties of B_4_C ceramics. When the sample is under the same sintering parameters—that is, the applied pressure is 50 MPa, the sintering temperature is 1900 °C, and the holding time is 10 min—the results are as follows: the density, Vickers hardness, and toughness of A-B_4_C are 93.15%, 29.6 ± 0.15 GPa, and 2.8 ± 0.06 MPa·m^1/2^, respectively. The density, Vickers hardness, and toughness of B-B_4_C are 98.29%, 32.84 ± 0.32 GPa, and 3.43 ± 0.11 MPa·m^1/2^, respectively. The density, Vickers hardness, and toughness of B_4_C are 94.75%, 30.87 ± 0.96 GPa, and 2.87 ± 0.11 MPa·m^1/2^, respectively.

Based on the discussion of the effect of the raw powders’ particle size on the sintering density and mechanical properties, the following conclusions can be reached. When the particle size is larger (d_50_ = 1.47 μm), the required sintering temperature is higher and the mechanical properties are poor. This is mainly because of the larger grain size (2 μm) of the sintered ceramic. For A-B_4_C powder, due to the extremely small size of the powder, the particles are prone to agglomeration, which is more unfavorable for sintering. Therefore, it is difficult to sinter densely, which leads to poor mechanical properties. In summary, the powder with d_50_ = 0.406 μm is a more suitable starting particle to achieve higher density and better mechanical properties at lower temperatures.

### 3.2. TEM Characterization

**Figure 13:**
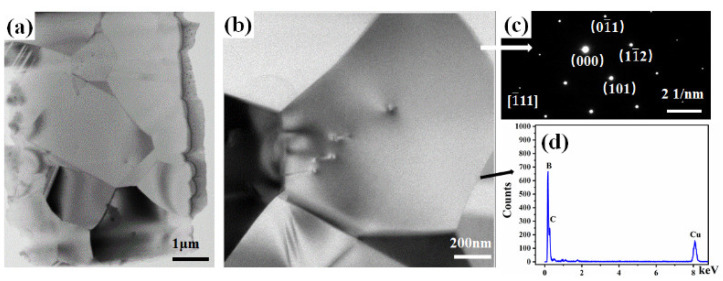
TEM images of B_4_C ceramics (**a**,**b**), the corresponding electron diffraction patterns (**c**), and EDS images of B_4_C ceramics (**d**).

Figure 13a,b illustrates the TEM images of B_4_C ceramics fabricated at 1950 °C. The straight and sharp grain boundaries are observed. The grain boundary conditions and grain size are consistent with the SEM analysis. The selected area electron diffraction image was observed: the [$\bar{1}11$] zone axis diffraction spots of B_4_C (Figure 13c). The EDS analysis of the grain boundary shows the peaks of B, C, and Cu, as illustrated in Figure 13d. The existence of Cu owes to the Cu grid that supports the B_4_C ceramics. There is no evidence of the existence of other elements.

**Figure 14:**
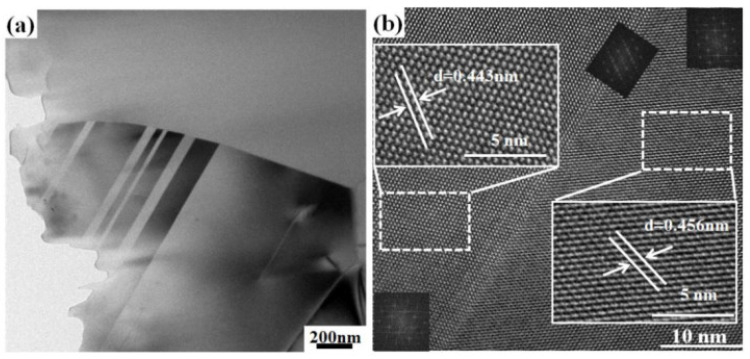
TEM image of B_4_C ceramic with twin structures (**a**) and the corresponding HRTEM image (**b**).

A TEM image of twin structures of B_4_C ceramic is illustrated in Figure 14a. The twin structures in the B_4_C ceramics can be regarded as sub-grain boundaries. This structure can obstruct the crack propagation, thereby enhancing the fracture toughness of the ceramics [32,33,34]. Sairam et al. [22] reported that the twin structures in SPS-sintered B_4_C ceramics are significantly higher than those in hot-pressed B_4_C ceramics. The combined action of the force field and electric field of SPS makes matter easier to rearrange and deform, which is conducive to the formation of twin structures. The twin structures have a relatively low energy interface of the grain boundaries and were believed to act as an obstacle to the crack displacement. The typical HRTEM image of a twin structure is illustrated in Figure 14b. The twin boundaries have a width of less than 1 nm, and the interplanar distance between the two sides of the twin boundaries are significantly different, 0.443 and 0.456 nm, respectively. Based on the EDS analysis and the HRTEM image, the grain boundaries of SPS produced B_4_C ceramics are clear and clean, and no second phase is observed. This is necessary for the ceramic to hold high flexural strength [35].

## 4. Conclusions

Near completely dense B_4_C ceramics were fabricated by SPS without any additives. The influence of sintering parameters and the particle size of the raw material on the mechanical performance and microstructure of pure B_4_C samples was discussed. It was found that denser B_4_C ceramics were achieved at lower temperatures (1850 °C) and a short holding time of 10 min with an applied pressure of 50 MPa. The B_4_C ceramic using a medium particle size of 0.406 μm exhibited excellent mechanical properties. The Vickers hardness of near theoretically dense B_4_C ceramics was 33.5 ± 0.2 GPa and the toughness was 3.21 ± 0.13 MPa·m^1/2^. As shown in the EBSD results, the grain size of B_4_C ceramics was in the range of 1 to 5 μm and the average grain size was about 1.4 μm. No grain coarsening was observed in the B_4_C ceramics prepared by SPS. The microstructural observation showed that there were twin structures in the samples, which is one of the reasons why SPS-sintered ceramic can obtain excellent properties.

## Figures and Tables

**Table 1:** The Vickers hardness and toughness of B_4_C ceramics with different sintering processes.

Article	Powder Particle Size (μm)	Method: Pressure; Temperature; Heating Rate; Soaking Time	Fracture Toughness (MPa·m^1/2^); Vickers Hardness (GPa)
Ref. [18]	1.21 μm	HPS: 40 MPa; 1900 °C; 10 °C/min; 60 min	2.76 ± 0.25; 21.8 ± 1.1
		SPS: 40 MPa; 1900 °C; 100 °C/min; 6 min	3.15 ± 0.23; 26.4 ± 4.3
Ref. [21]	2.4 μm	SPS: 50 MPa; 1800 °C; 100 °C/min; 15 min	2.8; 37.2
Ref. [23]	0.5 μm	SPS: 75 MPa; 1800 °C; 100 °C/min; 15 min	2.5 ± 0.2; 34 ± 2
Ref. [30]	0.3 μm	PSPS: 0 MPa; 2100 °C; 100 °C/min; 5 min	-; 27
Our work	0.5 μm	SPS: 50 MPa; 1850 °C; 100 °C/min; 10 min	3.21 ± 0.13; 33.5 ± 0.2

## Data Availability

The data presented in this study are available on request from the corresponding author.
